# Bidirectional Associations Between Cardiometabolic Multimorbidity and Depression and Mediation of Lifestyles: A Multicohort Study

**DOI:** 10.1016/j.jacasi.2024.06.004

**Published:** 2024-08-13

**Authors:** Yaguan Zhou, Mika Kivimäki, Carmen C.W. Lim, Rodrigo M. Carrillo-Larco, Shige Qi, Xifeng Wu, Xiaolin Xu

**Affiliations:** a School of Public Health, The Second Affiliated Hospital, Zhejiang University School of Medicine, Hangzhou, Zhejiang, China; b The Key Laboratory of Intelligent Preventive Medicine of Zhejiang Province, Hangzhou, Zhejiang, China; c UCL Brain Sciences, University College London, London, United Kingdom; d National Centre for Youth Substance Use Research, Faculty of Health and Behavioral Sciences, The University of Queensland, Brisbane, Queensland, Australia; e School of Psychology, Faculty of Health and Behavioral Sciences, The University of Queensland, Brisbane, Queensland, Australia; f Emory Global Diabetes Research Center, Emory University, Atlanta, Georgia, USA; g Hubert Department of Global Health, Rollins School of Public Health, Emory University, Atlanta, Georgia, USA; h National Center for Chronic and Noncommunicable Disease Control and Prevention, Chinese Center for Disease Control and Prevention, Beijing, China; i School of Public Health, Faculty of Medicine, The University of Queensland, Brisbane, Queensland, Australia

**Keywords:** cardiovascular diseases, diabetes, lifestyle modifications, mental health, reciprocal relationship

## Abstract

**Background:**

Cardiometabolic multimorbidity (CMM) and depression are major health concerns, and the onset of either condition may heighten the risk of developing the other.

**Objectives:**

The goal of this study was to characterize the reciprocal associations between CMM and depression among middle-aged and older adults.

**Methods:**

This multicohort study used harmonized data from 5 prospective cohorts from China, South Korea, the United States, the United Kingdom, and Europe. Cardiometabolic diseases (CMDs) (including diabetes, heart diseases, and stroke) and depression were assessed at baseline and at 7 to 8 years’ follow-up. Lifestyle factors, including physical activity, alcohol consumption, and smoking status, were regarded as potential mediators. Two sets of analyses, CMM-depression analyses (n = 67,188) and depression-CMM analyses (n = 65,738), were conducted to explore the bidirectional associations between CMM and depression.

**Results:**

In the CMM-depression analyses, 16,596 (24.7%) individuals developed depression. Participants with a single CMD (HR: 1.24; 95% CI:1.19-1.29) and CMM (HR: 1.52; 95% CI: 1.42-1.63) at baseline had higher risks of depression occurring. Physical activity and alcohol consumption significantly mediated 7.5% and 6.9% of the CMM-depression association, respectively. In the depression-CMM analyses, 1,461 (2.2%) participants developed CMM. The HR for developing CMM was 1.31 (95% CI: 1.14-1.50) in patients with depression, with increased risk of developing more CMDs. Physical activity and alcohol consumption mediated 12.0% and 7.1% of the depression-CMM association. The bidirectional relationships were more pronounced in Western countries than in Asian countries.

**Conclusions:**

CMM and depression were bidirectionally associated. The mediated effects of lifestyle factors were larger in the depression-lifestyle-CMM pathway than in the CMM-lifestyle-depression pathway.

Depression, a common and debilitating psychiatric disorder, affects approximately 6% of the global population.^1^^,^^2^ In 2017, the World Health Organization listed depression as a leading cause of disability worldwide.^3^ In addition to its psychological impact, depression is often accompanied by somatic conditions^4^ such as dyslipidemia, rheumatoid arthritis, sleep disorders, and cardiometabolic diseases (CMDs), including diabetes, heart diseases, and stroke.^5^, ^6^, ^7^ Recent trends, including population aging, increased life expectancy, and lifestyle changes, have contributed to the accumulation of chronic conditions, leading to the increase in multimorbidity.^8^ In particular, there has been significant research interest in cardiometabolic multimorbidity (CMM), which is defined as the co-existence of two or more CMDs.^9^ CMM increases the risk of activity limitation, cognitive dysfunction, dementia, and all-cause mortality,^10^^,^^11^ presenting an ongoing challenge to global health care systems.

Recent research has provided evidence on the relationship between depression and CMDs. A prospective study with 27-year follow-up found that depression increased the risk of cardiometabolic health problems (eg, diabetes, erectile dysfunction, sleep apnea).^12^ This finding was supported by two other cohort studies.^13^^,^^14^ The longitudinal association of CMDs with later depression has also been explored. For example, diabetes was found to be associated with increased risk of developing depression,^15^ and patients with stroke had a higher incidence of depression after hospitalization.^16^ However, these studies focused on single CMDs only, and, to the best of our knowledge, no current study has shed light directly on the reciprocal associations between depression and CMM. A population-based cohort study of 93,076 adults examined the bidirectional associations between depression and cardiovascular diseases (ischemic heart disease and stroke), but this study did not include diabetes and was exclusively conducted in Denmark, thus limiting the generalizability of its findings.^17^

CMM and depression are long-lasting and progressive, both of which could be partly attributable to modifiable indicators (eg, lifestyle factors).^18^^,^^19^ However, whether their bidirectional associations could be mediated by lifestyle factors remains unknown. To provide valuable insights for strategies to prevent and manage these significant physical and mental health challenges in the population,^20^ we leveraged harmonized data across 19 countries from Asia, North America, and Europe to generate robust evidence on the bidirectional associations between CMM and depression and the mediation effects of lifestyle factors among middle-aged and older adults.

## Methods

### Study Design and Participants

This prospective multicohort study adhered to the Strengthening the Reporting of Observational Studies in Epidemiology reporting guideline. Data were pooled across 19 countries from 5 sister cohort studies from the Program on Global Aging, Health and Policy^21^: CHARLS (China Health and Retirement Longitudinal Study); KLoSA (Korean Longitudinal Study of Aging); HRS (U.S. Health and Retirement Study); ELSA (English Longitudinal Study on Ageing); and SHARE (Survey of Health, Ageing and Retirement in Europe). These studies followed similar protocols and administered surveys every 2 or 3 years, therefore allowing meaningful cross-study comparisons. Additional details for each study have been described elsewhere.^22^, ^23^, ^24^, ^25^, ^26^

We used data from 4 waves of a consistent time period in each study, including waves 1 to 4 of CHARLS (2011-2019), waves 4 to 7 of KLoSA (2012-2019), waves 11 to 14 of HRS (2012-2019), waves 6 to 9 of ELSA (2012-2019), and waves 5 to 8 of SHARE (2013-2020) (Supplemental Table 1). In our analyses, we included participants if they were middle-aged and older adults (aged ≥45 years) and reported complete information on CMDs and depression at baseline. Participants aged >85 years at baseline were excluded to minimize survival bias.

This study has been exempt from ethical review because it was a secondary analysis of a public data set.

### CMDs and CMM

The range of CMDs included self-reported diabetes, heart diseases, and stroke,^27^ with definitions slight varied across studies. Heart diseases mainly referred to heart attack, coronary heart disease, angina, congestive heart failure, and other heart problems. The definition of stroke included ischemic attack in KLoSA and HRS; in ELSA and SHARE, it included cerebrovascular diseases. CMM was identified as the coexistence of ≥2 CMDs in an individual. In analyses of bidirectional associations, 2 variables were used: CMM status (no and yes) and CMD status (free of CMD, single CMD, and CMM). We additionally examined the number (0, 1, 2, and 3 CMDs) and combinations of CMDs, including the following groups: 1) none; 2) diabetes; 3) heart diseases; 4) stroke; 5) diabetes and heart diseases; 6) heart diseases and stroke; 7) diabetes and stroke; and 8) diabetes, heart diseases, and stroke.

### Depression

In CHARLS and KLoSA, depression was assessed by using the 10-item version of the Center for Epidemiologic Studies Depression Scale. In HRS and ELSA, depression was assessed by using the 8-item version of the Center for Epidemiologic Studies Depression Scale. In SHARE, depression was assessed by using the 12-item version of the EURO-D. Individuals were then dichotomized into 2 groups of non-depression and depression using predefined study-specific cutoff values.^28^, ^29^, ^30^, ^31^, ^32^ Detailed description of the assessment in each study is provided in Supplemental Table 2.

### Covariates

Covariates, including demographic characteristics (age, sex, and study), socioeconomic status (marital status, educational level, and total household wealth), lifestyle factors (smoking status, drinking status, physical activity, and body mass index), history of chronic conditions (hypertension, cancer, and lung diseases), and medication of chronic conditions (hypertension and lung diseases) were assessed. Lifestyle factors and medication of chronic conditions were regarded as time-dependent variables, according to previous research and empirical evidence.^33^ Total household wealth was calculated as the sum of all wealth components, including residence, business, vehicles, and savings account, excluding other debt or loans, and was categorized into quartiles. Further details of the measurement and harmonization of the covariates in each study are provided in Supplemental Table 2.

### Statistical Analysis

The baseline characteristics of participants are described as number (percentage) according to CMD status and depression at baseline, and chi-square tests were used to compare the between-group differences. Two sets of analyses, CMM-depression analyses and depression-CMM analyses, were conducted to explore the bidirectional associations between CMM and depression.

#### CMM-Depression Analyses

Cox regression models with time-dependent covariates were used to estimate the HRs and 95% CIs of the association between baseline CMM and depression incidence during follow-up. Participants were censored at interview month of depression or death, or end of follow-up of each cohort, whichever came first. Individuals were recorded as incident depression events if the depressive symptoms assessed by using study-specific instruments first appeared during follow-up. Considering the repeated measures on the same participants, we also applied generalized estimating equations (GEEs) for Poisson regression to estimate relative risks (RRs) and 95% CIs of the CMM-depression association. The mediation effects of lifestyles were further examined by using the mediation package in R (R Foundation for Statistical Computing). Physical activity, smoking status, and alcohol consumption were set as the mediators to estimate the total, indirect, and direct effects of CMM on depression, with the proportion mediated calculated via dividing indirect effect by total effect. Subgroup analyses were performed to compare the association of baseline CMM with incidence of depression among participants with different age (<65 years vs ≥65 years), sex, educational level, total household income, and studies. Considering the heterogeneity across studies, we conducted meta-analyses using random-effects models to summarize study-specific estimates for the CMM-depression association among the whole sample and sex-stratified samples, with the between-study heterogeneity tested using *I*^2^ statistics. The multivariable logistic regression models were conducted to examine ORs and 95% CIs of the association between CMM at baseline and depression trajectory during follow-up, using a group-based trajectory model implemented with the PROC TRAJ procedure in SAS (Supplemental Methods).

#### Depression-CMM Analyses

Cox regression models with time-dependent covariates and the GEEs for Poisson regression were conducted to estimate HRs, RRs, and 95% CIs of the association between depression and CMM incidence. In the Cox regression models, participants were censored at interview month of CMM or death, or end of follow-up of each cohort, whichever came first. Individuals were recorded as incident CMM events if they reported to have CMM for the first time. Correspondingly, the mediation effects of lifestyles (physical activity, smoking status, and alcohol consumption) for the depression-CMM analyses were further examined. Subgroup analyses and meta-analyses using random-effects models for the association between depression and CMM incidence were also performed.

All tests in this study were 2-sided with a significance level of *P* < 0.05. Statistical analyses were conducted by using SAS version 9.4 (SAS Institute, Inc) and R version 4.2.2.

## Results

### Baseline Characteristics

**Figure 1:**
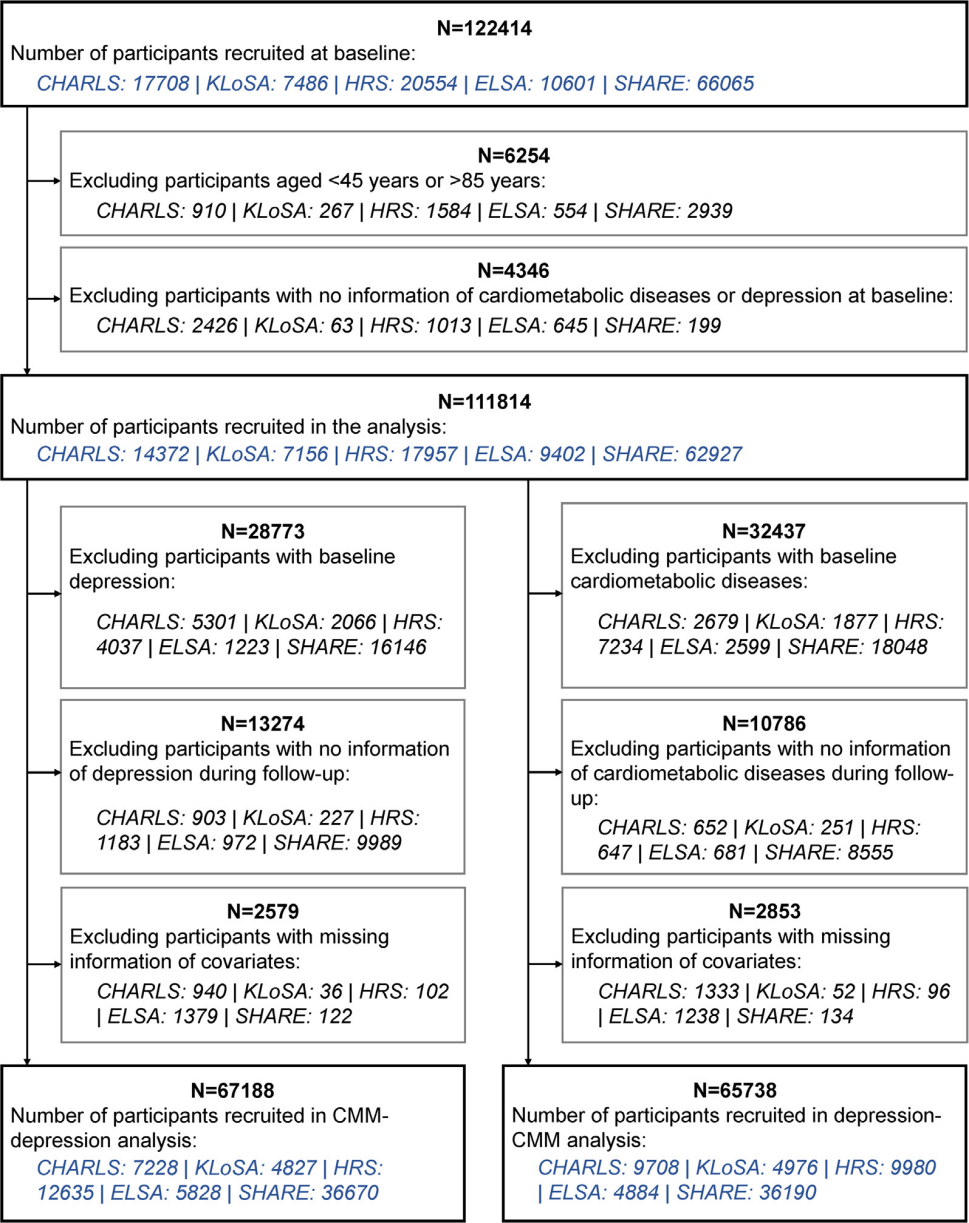
Flow Diagram of Participant Selection Participants were selected according to the inclusion and exclusion criteria, yielding 2 sample sets for the bidirectional analyses. CHARLS = China Health and Retirement Longitudinal Study; CMM = cardiometabolic multimorbidity; ELSA = English Longitudinal Study on Ageing; HRS = U.S. Health and Retirement Study; KLoSA = Korean Longitudinal Study of Aging; SHARE = Survey of Health, Ageing and Retirement in Europe.

A total of 111,814 participants provided information on CMDs and depression at baseline. Participants already with depression at baseline (n = 28,773), lacking information on depression during follow-up (n = 13,274), or with missing information of covariates (n = 2,579) were excluded, yielding the final sample of 67,188 individuals for the CMM-depression analyses. The mean age of participants in the CMM-depression analyses was 64.36 ± 9.22 years, and 35,243 (52.5%) were female. Of these, 7,228 participants were from China, 4,827 from South Korea, 12,635 participants were from the United States, and 42,498 from the United Kingdom and 15 other European countries (Figure 1, Supplemental Table 1). At baseline, 49,892 (74.3%) of 67,188 individuals did not have any CMDs, 14,082 (21.0%) had a single CMD, and 3,214 (4.8%) had CMM. Table 1 shows the baseline characteristics of participants in the CMM-depression analyses according to CMD status.

**Table 1:** Baseline Characteristics of Participants Included in the CMM-Depression Analyses According to CMD Status

	Total (N = 67,188)	CMD Status at Baseline			Chi-Square	*P* Value
		None (n = 49,892)	Single CMD (n = 14,082)	CMM (n = 3,214)		
Study					1,434.75	<0.001
CHARLS	7,228 (10.8%)	6,195 (12.4%)	901 (6.4%)	132 (4.1%)		
KLoSA	4,827 (7.2%)	3,794 (7.6%)	890 (6.3%)	143 (4.4%)		
HRS	12,635 (18.8%)	8,045 (16.1%)	3,533 (25.1%)	1,057 (32.9%)		
ELSA	5,828 (8.7%)	4,361 (8.7%)	1,276 (9.1%)	191 (5.9%)		
SHARE	36,670 (54.6%)	27,497 (55.1%)	7,482 (53.1%)	1,691 (52.6%)		
Age, y					3,867.40	<0.001
45-54	10,556 (15.7%)	9,374 (18.8%)	1,054 (7.5%)	128 (4.0%)		
55-64	25,054 (37.3%)	20,138 (40.4%)	4,175 (29.6%)	741 (23.1%)		
65-74	20,539 (30.6%)	14,080 (28.2%)	5,199 (36.9%)	1,260 (39.2%)		
≥75	11,039 (16.4%)	6,300 (12.6%)	3,654 (25.9%)	1,085 (33.8%)		
Sex					377.79	<0.001
Male	31,945 (47.5%)	22,634 (45.4%)	7,506 (53.3%)	1,805 (56.2%)		
Female	35,243 (52.5%)	27,258 (54.6%)	6,576 (46.7%)	1,409 (43.8%)		
Education level					44.58	<0.001
Primary	22,926 (34.1%)	16,732 (33.5%)	4,955 (35.2%)	1,239 (38.6%)		
Secondary	26,127 (38.9%)	19,526 (39.1%)	5,409 (38.4%)	1,192 (37.1%)		
Tertiary	18,135 (27.0%)	13,634 (27.3%)	3,718 (26.4%)	783 (24.4%)		
Total household wealth^a^					621.61	<0.001
Q1 (lowest)	16,281 (25.0%)	11,071 (23.0%)	4,085 (29.5%)	1,125 (35.3%)		
Q2	16,351 (25.1%)	11,910 (24.7%)	3,596 (26.0%)	845 (26.5%)		
Q3	16,295 (25.0%)	12,328 (25.6%)	3,287 (23.8%)	680 (21.3%)		
Q4 (highest)	16,306 (25.0%)	12,911 (26.8%)	2,859 (20.7%)	536 (16.8%)		
Marital status					426.87	<0.001
Married or partnered	52,791 (78.6%)	39,903 (80.0%)	10,588 (75.2%)	2,300 (71.7%)		
Separated or divorced	5,035 (7.5%)	3,635 (7.3%)	1,138 (8.1%)	262 (8.2%)		
Widowed	6,846 (10.2%)	4,429 (8.9%)	1,883 (13.4%)	534 (16.6%)		
Never married	2,484 (3.7%)	1,904 (3.8%)	466 (3.3%)	114 (3.6%)		
Physical activity					529.61	<0.001
No	8,497 (13.4%)	5,528 (11.9%)	2,189 (16.1%)	780 (24.8%)		
Yes	54,710 (86.6%)	40,958 (88.1%)	11,390 (83.9%)	2,362 (75.2%)		
Alcohol assumption					410.87	<0.001
Less than weekly drinking	36,399 (54.9%)	25,972 (52.8%)	8,264 (59.3%)	2,163 (67.8%)		
Weekly drinking or more	29,930 (45.1%)	23,223 (47.2%)	5,682 (40.7%)	1,025 (32.2%)		
Current smoking					162.48	<0.001
No	55,349 (82.4%)	40,559 (81.3%)	11,998 (85.2%)	2,792 (86.9%)		
Yes	11,834 (17.6%)	9,329 (18.7%)	2,084 (14.8%)	421 (13.1%)		
Body mass index, kg/m^2^					2,208.95	<0.001
<18.5	958 (1.5%)	802 (1.7%)	142 (1.0%)	14 (0.4%)		
18.5-24.9	23,780 (36.5%)	19,602 (40.6%)	3,580 (26.2%)	598 (19.2%)		
25-29.9	25,525 (39.2%)	18,709 (38.7%)	5,613 (41.0%)	1,203 (38.6%)		
≥30.0	14,849 (22.8%)	9,206 (19.1%)	4,341 (31.7%)	1,302 (41.8%)		
Hypertension					5,101.87	<0.001
No	38,712 (57.6%)	32,638 (65.4%)	5,359 (38.1%)	715 (22.2%)		
Yes	28,460 (42.4%)	17,239 (34.6%)	8,722 (61.9%)	2,499 (77.8%)		
Cancer					313.60	<0.001
No	61,867 (92.1%)	46,464 (93.1%)	12,607 (89.5%)	2,796 (87.0%)		
Yes	5,313 (7.9%)	3,424 (6.9%)	1,472 (10.5%)	417 (13.0%)		
Lung diseases					492.44	<0.001
No	62,946 (93.7%)	47,291 (94.8%)	12,868 (91.4%)	2,787 (86.7%)		
Yes	4,233 (6.3%)	2,595 (5.2%)	1,212 (8.6%)	426 (13.3%)		
Medication for hypertension					4,879.99	<0.001
No	42,787 (63.8%)	35,456 (71.2%)	6,393 (45.5%)	938 (29.3%)		
Yes	24,233 (36.2%)	14,314 (28.8%)	7,657 (54.5%)	2,262 (70.7%)		
Medication for lung disease					231.69	<0.001
No	65,584 (97.7%)	48,955 (98.2%)	13,582 (96.6%)	3,047 (95.0%)		
Yes	1,552 (2.3%)	909 (1.8%)	484 (3.4%)	159 (5.0%)		

Values are n (%) unless otherwise indicated.

CHARLS = China Health and Retirement Longitudinal Study; CMD = cardiometabolic disease; CMM = cardiometabolic multimorbidity; ELSA = English Longitudinal Study on Ageing; HRS = U.S. Health and Retirement Study; KLoSA = Korean Longitudinal Study of Aging; Q = quartile; SHARE = Survey of Health, Ageing and Retirement in Europe.

a Total household wealth was categorized into quartiles.

Similarly, for the depression-CMM analyses, this study excluded participants already with any CMDs at baseline (n = 32,437), lacking information on CMDs during follow-up (n = 10,786), or with missing information of covariates (n = 2,853), leaving 65,738 participants in the analyses. Their mean age was 63.02 ± 9.13 years, and 37,970 (57.8%) were female. There were 9,708 individuals from China, 4,976 from South Korea, 9,980 individuals from the United States, and 41,074 from the United Kingdom, and 15 from other European countries (Figure 1, Supplemental Table 1). A total of 14,673 (22.3%) participants had depression at baseline; Table 2 shows the baseline characteristics of participants in the depression-CMM analyses according to depression status.

**Table 2:** Baseline Characteristics of Participants Included in the Depression-CMM Analyses According to Depression Status

	Total (N = 65,738)	Depression at Baseline		Chi-Square	*P* Value
		No (n = 51,065)	Yes (n = 14,673)		
Study				648.37	<0.001
CHARLS	9,708 (14.8%)	6,348 (12.4%)	3,360 (22.9%)		
KLoSA	4,976 (7.6%)	3,794 (7.4%)	1,182 (8.1%)		
HRS	9,980 (15.2%)	8,146 (16.0%)	1,834 (12.5%)		
ELSA	4,884 (7.4%)	4,392 (8.6%)	492 (3.4%)		
SHARE	36,190 (55.1%)	28,385 (55.6%)	7,805 (53.2%)		
Age, y				62.45	<0.001
45-54	12,753 (19.4%)	9,623 (18.8%)	3,130 (21.3%)		
55-64	26,274 (40.0%)	20,574 (40.3%)	5,700 (38.8%)		
65-74	18,017 (27.4%)	14,376 (28.2%)	36,41 (24.8%)		
≥75	8,694 (13.2%)	6,492 (12.7%)	2,202 (15.0%)		
Sex				535.75	<0.001
Male	27,768 (42.2%)	23,211 (45.5%)	4,557 (31.1%)		
Female	37,970 (57.8%)	27,854 (54.5%)	10,116 (68.9%)		
Education level				660.38	<0.001
Primary	24,569 (37.4%)	17,237 (33.8%)	7,332 (50.0%)		
Secondary	24,763 (37.7%)	19,917 (39.0%)	4,846 (33.0%)		
Tertiary	16,406 (25.0%)	13,911 (27.2%)	2,495 (17.0%)		
Total household wealth^a^				632.53	<0.001
Q1 (lowest)	15,784 (24.9%)	11,058 (22.4%)	4,726 (33.9%)		
Q2	15,829 (25.0%)	12,205 (24.7%)	3,624 (26.0%)		
Q3	15,857 (25.1%)	12,750 (25.8%)	3,107 (22.3%)		
Q4 (highest)	15,830 (25.0%)	13,355 (27.1%)	2,475 (17.8%)		
Marital status				337.07	<0.001
Married or partnered	51,339 (78.1%)	40,837 (80.0%)	10,502 (71.6%)		
Separated or divorced	5,046 (7.7%)	3,700 (7.2%)	1,346 (9.2%)		
Widowed	6,748 (10.3%)	4,548 (8.9%)	2,200 (15.0%)		
Never married	2,580 (3.9%)	1,958 (3.8%)	622 (4.2%)		
Physical activity				398.46	<0.001
No	8,432 (14.0%)	5,649 (11.9%)	2,783 (21.7%)		
Yes	51,955 (86.0%)	41,937 (88.1%)	10,018 (78.3%)		
Alcohol assumption				409.26	<0.001
Less than weekly drinking	36,275 (56.0%)	26,651 (52.9%)	9,624 (66.5%)		
Weekly drinking or more	28,555 (44.0%)	23,710 (47.1%)	4,845 (33.5%)		
Current smoking				119.01	<0.001
No	52,656 (80.1%)	41,448 (81.2%)	11,208 (76.4%)		
Yes	13,076 (19.9%)	9,613 (18.8%)	3,463 (23.6%)		
Body mass index, kg/m^2^				145.18	<0.001
<18.5	1,358 (2.1%)	824 (1.7%)	534 (3.8%)		
18.5-24.9	26,247 (41.3%)	20,109 (40.7%)	6,138 (43.8%)		
25-29.9	23,811 (37.5%)	19,158 (38.7%)	4,653 (33.2%)		
≥30.0	12,074 (19.0%)	9,377 (19.0%)	2,697 (19.2%)		
Hypertension				37.15	<0.001
No	42,542 (64.7%)	33,417 (65.5%)	9,125 (62.2%)		
Yes	23,166 (35.3%)	17,632 (34.5%)	5,534 (37.8%)		
Cancer				9.53	<0.001
No	61,119 (93.0%)	47,578 (93.2%)	13,541 (92.3%)		
Yes	4,610 (7.0%)	3,483 (6.8%)	1,127 (7.7%)		
Lung diseases				193.41	<0.001
No	61,636 (93.8%)	48,406 (94.8%)	13,230 (90.2%)		
Yes	4,095 (6.2%)	2,653 (5.2%)	1,442 (9.8%)		
Medication for hypertension				9.73	0.001
No	46,536 (71.0%)	36,307 (71.3%)	10,229 (69.9%)		
Yes	19,042 (29.0%)	14,631 (28.7%)	4,411 (30.1%)		
Medication for lung disease				103.10	<0.001
No	64,204 (97.7%)	50,107 (98.2%)	14,097 (96.1%)		
Yes	1,498 (2.3%)	929 (1.8%)	569 (3.9%)		

Values are n (%).

Abbreviations as in Table 1.

a Total household wealth was categorized into quartiles.

### CMM-Depression Analyses: the Association Between CMM and Incidence of Depression

**Figure 2:**
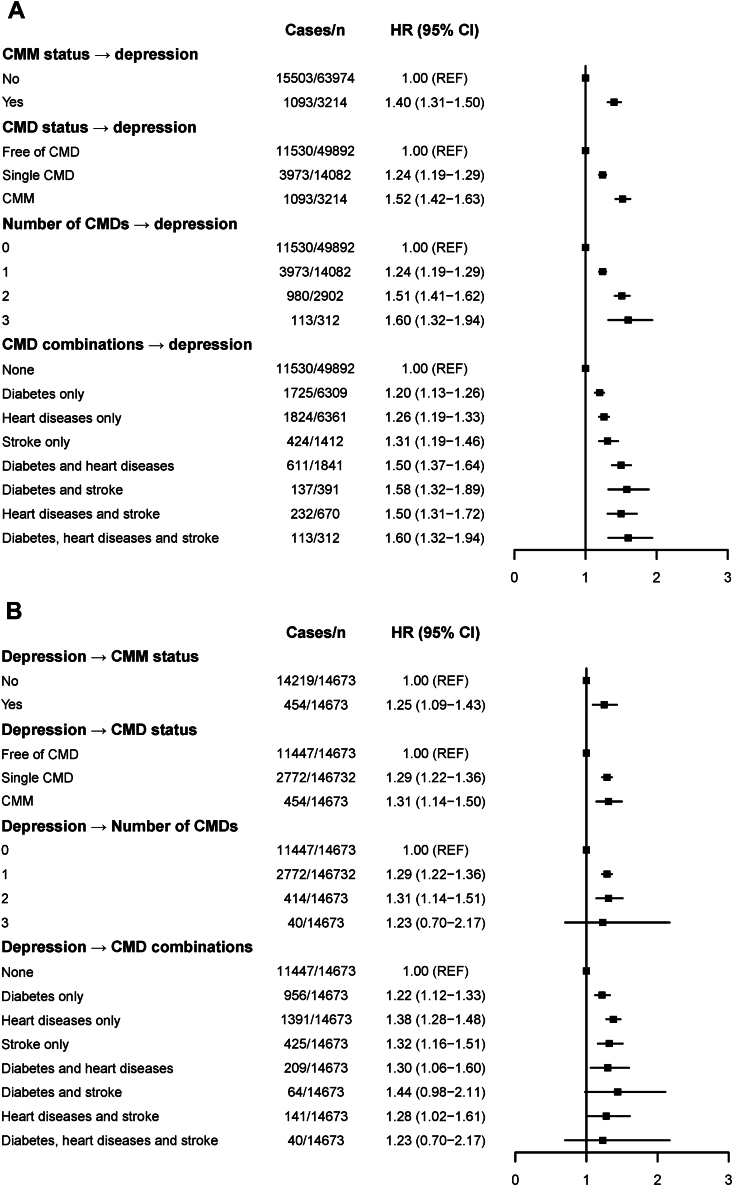
Associations Between CMM and Depression The forest plots show the HRs and 95% CIs for the cardiometabolic multimorbidity (CMM)-depression analyses (A) and the depression-CMM analyses (B) generated by Cox regression models with time-dependent covariates. Models were adjusted for age, sex, and study, marital status, educational level, and total household wealth, smoking status, drinking status, physical activity, body mass index (BMI), history of hypertension, cancer, and lung diseases, and medication of hypertension and lung diseases. CMD = cardiometabolic disease.

Of the 67,188 participants in the CMM-depression analyses, 16,596 (24.7%) progressed to depression during follow-up. Figure 2A displays the association between baseline CMM and depression incidence during follow-up using Cox regression models. Individuals with a single CMD (HR: 1.24; 95% CI: 1.19-1.29) and CMM (HR: 1.52; 95% CI: 1.42-1.63) at baseline had higher risks of developing depression. The risk of depression incidence rose as the number of baseline CMDs increased, with HRs ranging from 1.24 (95% CI: 1.19-1.29) to 1.60 (95% CI: 1.32-1.94). Those with combinations of diabetes, heart diseases, and stroke had the highest risk of developing depression (HR: 1.60; 95% CI: 1.32-1.94) (Central Illustration). Results from the GEE analyses also showed an increased risk of depression in those with a single CMD (RR: 1.34; 95% CI: 1.29-1.40) and CMM (RR: 1.78; 95% CI: 1.68-1.89), after adjusting for covariates (Supplemental Table 3).

**Central Illustration:**
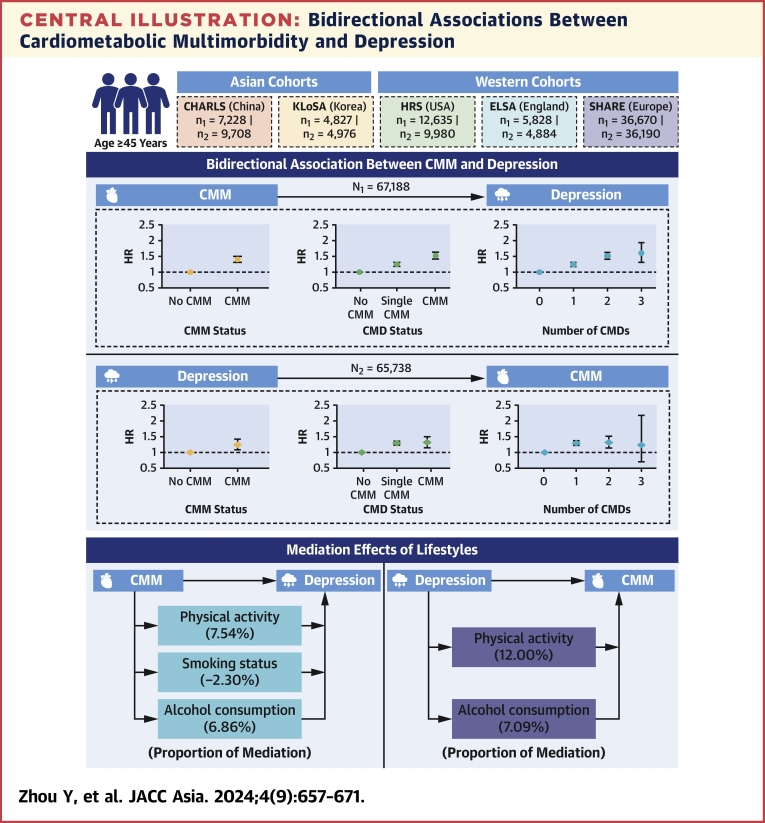
Bidirectional Associations Between Cardiometabolic Multimorbidity and Depression Participants aged ≥45 years from 5 cohorts in Asian and Western countries were included to evaluate the bidirectional associations between cardiometabolic multimorbidity (CMM) and depression, and the mediation effects of lifestyles. CHARLS = China Health and Retirement Longitudinal Study; CMD = cardiometabolic disease; ELSA = English Longitudinal Study on Ageing; HRS = the U.S. Health and Retirement Study; KLoSA = Korean Longitudinal Study of Aging; SHARE = Survey of Health, Ageing and Retirement in Europe.

**Figure 3:**
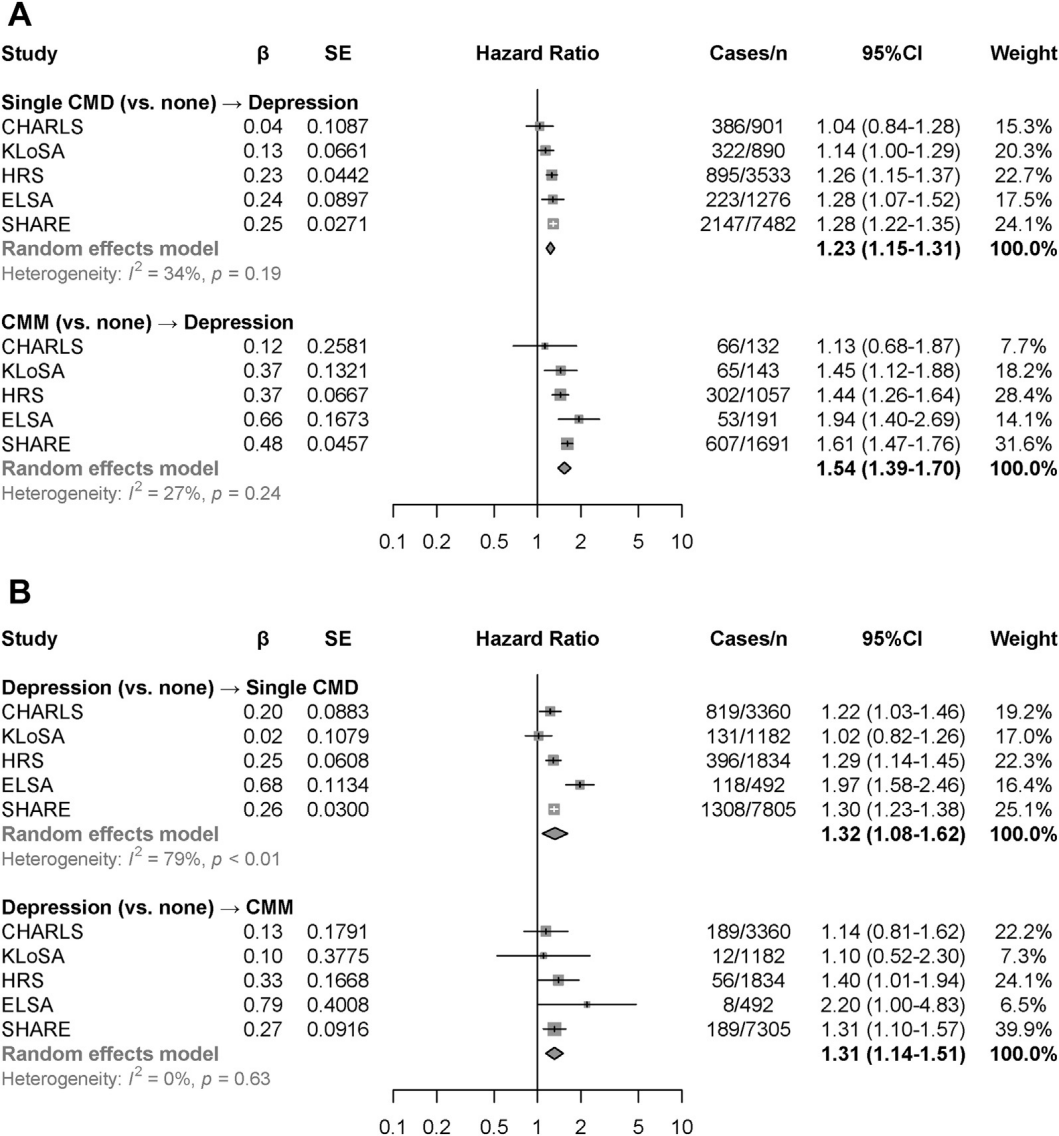
Meta-Analyses of the Associations Between CMM and Depression Random-effects meta-analyses were conducted to calculate the HRs and 95% CIs for CMM-depression analyses (A) and depression-CMM analyses (B). Heterogeneity was tested by using *I*^2^ statistics. Abbreviations as in Figures 1 and 2.

The mediation effects of lifestyle factors on the association of CMM with depression are presented in Table 3. Physical activity and alcohol consumption positively mediated the CMM-depression association (mediation proportions of physical activity: 7.5%; of alcohol consumption: 6.9%), while smoking status had a negative mediation effect (mediation proportion: –2.3%). Subgroup analyses according to age, sex, educational level, and total household income yielded broadly similar results (Supplemental Tables 4 to 6). The CMM-depression association from Cox regression models were stronger in Western countries than in Asian countries (Figure 3A), with no sex differences observed (Supplemental Figures 1A to 1B). The pooled HRs for depression incidence were 1.23 (single CMD vs none; 95% CI: 1.15-1.31) and 1.54 (CMM vs none; 95% CI: 1.39-1.70), with mild between-study heterogeneity observed (*I*^2^ = 27%-34%). The pooled RRs for depression incidence from GEE analyses showed no heterogeneity (Supplemental Figure 2A). Three trajectories for depression symptoms were identified: “persistently symptom-free,” “persistently low,” and “increasing” (Supplemental Table 7, Supplemental Figure 3). Increased risk for the “persistently low” and “increasing” depression trajectories were observed among those with CMM (Supplemental Table 8).

**Table 3:** Mediation Effects of Lifestyle Factors on the Bidirectional Associations Between Cardiometabolic Multimorbidity and Depression

	Direct Effect		Indirect Effect		% Mediated
	Coefficients (95% CI)	*P* Value	Coefficients (95% CI)	*P* Value	
CMM-depression analyses					
CMD status → depression					
Physical activity	4.13 (2.86-5.41)	<0.001	0.34 (0.25-0.43)	<0.001	7.5
Smoking status	4.14 (2.18-6.24)	<0.001	−0.10 (−0.18 to −0.03)	0.006	−2.3
Alcohol consumption	4.39 (3.08-5.66)	<0.001	0.32 (0.24-0.42)	<0.001	6.9
Depression-CMM analyses					
Depression → single CMD					
Physical activity	7.13 (5.40-8.93)	<0.001	0.30 (0.18-0.44)	<0.001	4.0
Smoking status	7.37 (5.51-9.12)	<0.001	0.01 (−0.05 to 0.08)	0.77	1.1
Alcohol consumption	7.04 (5.53-8.61)	<0.001	0.25 (0.16-0.35)	<0.001	3.5
Depression → CMM					
Physical activity	2.50 (−1.43 to 6.64)	<0.001	0.40 (0.14-0.65)	<0.001	12.0
Smoking status	4.38 (1.49-7.18)	<0.001	−0.06 (−0.20 to 0.05)	0.29	−1.4
Alcohol consumption	4.06 (1.20-6.98)	0.006	0.31 (0.15-0.48)	<0.001	7.1

Models were adjusted for age, sex, study, marital status, educational level, total household wealth, body mass index (BMI), history of hypertension, cancer and lung diseases, and medication of hypertension and lung diseases.

Abbreviations as in Table 1.

### Depression-CMM Analyses: the Association Between Depression and Incidence of CMM

Of the 65,738 participants in the depression-CMM analyses, 10,082 (15.3%) participants developed a single CMD, and 1,461 (2.2%) developed CMM, including 103 (0.2%) with diabetes, heart diseases, and stroke. Figure 2B displays the association between baseline depression and incidence of CMM during follow-up using Cox regression models. Those with depression at baseline had higher risk of developing a single CMD (HR: 1.29; 95% CI: 1.22-1.36) and CMM (HR: 1.31; 95% CI: 1.14-1.50). Depression was associated with an elevated risk of developing more CMDs, although its association with 3 CMDs was imprecisely estimated. In addition, depression was significantly associated with specific combinations of CMDs (eg, HR: 1.30; 95% CI: 1.06-1.60 for diabetes and heart diseases) (Central Illustration). Results from GEE analyses yielded similar results, with RRs of 1.20 (95% CI: 1.14-1.26) and 1.53 (95% CI: 1.33-1.76) for developing a single CMD and CMM, respectively (Supplemental Table 3).

The mediation effects of lifestyle factors on the association of depression with CMM are presented in Table 3. Physical activity and alcohol consumption mediated the depression-CMM association, with mediation proportions of 12.0% and 7.1% respectively, while the mediation effect of smoking status was nonsignificant. The subgroups analyses showed that age, sex, educational level, and total household income did not substantially modify the depression-CMM association (Supplemental Tables 9 to 11). However, study-specific HRs indicated that the depression-CMM association attenuated to nonsignificance in Asian countries (eg, in CHARLS, HR for CMM: 1.10; 95% CI: 0.52-2.30) (Figure 3B), showing similar patterns between the sexes (Supplemental Figures 1C to 1D). The pooled HR was 1.32 (95% CI: 1.08-1.62: *I*^2^ = 79%) for developing a single CMD and 1.31 (95% CI: 1.14-1.51; *I*^2^ = 0%) for developing CMM among those with baseline depression (Figure 3B). The meta-analyses from GEEs for Poisson regression models also generated significant results (RR of developing a single CMD: 1.18 [95% CI: 1.12-1.25]; RR of developing CMM: 1.51 [95% CI: 1.31-1.74]), with between-study heterogeneity of 0% to 22% (Supplemental Figure 2B).

## Discussion

In this study involving 5 aging cohorts across 19 countries from Asia, North America, and Europe, bidirectional associations between CMM and depression were found. Individuals with a single CMD and CMM at baseline had an elevated risk of developing depression. Alcohol consumption and physical activity significantly mediated a proportion of 6.9% to 7.5% of the association of CMM with depression. Similarly, individuals with depression had an increased risk of developing CMM, which rose with the number of CMDs, though this association was imprecise in some analyses. The mediation proportion of alcohol consumption and physical activity was 7.1% to 12.0% for the depression-CMM association.

Although the association between CMDs and depression has been widely explored in prior research,^12^^,^^34^^,^^35^ little attention has been paid to the longitudinal impact of CMM on the incidence of depression. Our study addressed this knowledge gap and found that individuals with CMM, regardless of specific CMD combinations, experienced a heightened risk of developing depression. Considering that the variables were collected over time as repeated measures, we further conducted GEEs for Poisson regression to longitudinally estimate the CMM-depression association, the results of which suggest that the longitudinal association of CMM with depression is robust. Our meta-analyses and additional analyses examining depression trajectories during follow-up showed a similar pattern of results, with no significant between-study heterogeneity.

We also observed a longitudinal association between depression and subsequent CMM from both Cox regression models with time-dependent covariates and GEE analyses. This finding concurs with a cohort study based on the UK Biobank^14^ but that study included only UK participants, limiting the generalization of its results. By using a broader range of nationally representative samples from 19 countries, our study confirmed the relationship between depression and CMM incidence, although the HRs for some categories of CMM were imprecisely estimated (eg, CMM of 3 CMDs), probably owing to the small participant numbers in these categories. In our meta-analyses, the main associations between depression and CMM incidence were significant but with significant between-study heterogeneity. The study-specific HRs suggested that the longitudinal association between depression and CMM incidence might be more pronounced in Western countries than in Asian countries, stressing the importance of considering large-sample populations with different cultural contexts in future research. However, the meta-analyses using random-effects models from GEEs observed nonsignificant between-study heterogeneity.

Our findings further included 3 lifestyle factors and found the bidirectional associations between CMM and depression were positively mediated by physical activity and alcohol consumption, suggesting that targeted interventions limiting drinking and improving physical activity would be more effective. Smoking status was found to negatively mediate the CMM-depression association, which might be explained by the short follow-ups and reverse causality. In addition, larger mediated proportions of lifestyle factors were observed in the depression-CMM pathway than otherwise, suggesting that lifestyle interventions might be more effective to prevent or delay CMM onset among individuals with depression.

Our findings are biologically plausible. The observed associations of stroke, heart diseases, and diabetes with elevated depression incidence in this study are consistent with at least 3 mechanistic hypotheses: the poststroke depression hypothesis, the vascular depression hypothesis,^36^ and the diabetes-depression hypothesis.^37^^,^^38^ Poststroke depression refers to depression that occurs in the context of a clinically apparent stroke^36^ and might be related to inflammatory processes, hypothalamic-pituitary-adrenal axis abnormalities, and alterations in neuroplasticity and glutamate neurotransmission.^39^ Vascular depression, in turn, refers to depression in the presence of vascular risk factors accompanied by neuropsychological deficit and distinct localized brain pathology.^36^ The longitudinal association between diabetes and depression might be driven by vascular and metabolic pathology.^40^ Microvascular dysfunction is common in people with diabetes, including effects on the brain, and its diabetes-related drivers are hyperglycemia, obesity and insulin resistance, and hypertension.^37^^,^^38^

There are also several plausible mechanisms for the effects of depression on CMDs, although the evidence is less robust. The depression-CMD association might be explained by depression-related alterations in the autonomic nervous system, platelet receptors and function, coagulopathic factors, proinflammatory cytokines, and neurohormonal factors.^41^ Chronic stress, a correlate of depression, increases the production of cortisol in the adrenal cortex and activates the sympathetic nervous system,^42^^,^^43^ while hypercortisolemia and sympathetic nervous system activation further contribute to insulin resistance, diabetes, and metabolic syndrome.^44^ Hypercortisolemia can induce tachyphylaxis of the reward system and the disturbed neurogenesis in the hippocampus, both related to depression.^45^^,^^46^

Our research has important implications for clinical practice and public health policy. The evidence highlights the likelihood of shared pathways between physical and mental conditions, suggesting that preventive interventions should commence early.^47^ Health care providers should be attentive to the heightened risk of depression in patients with CMM and implement timely interventions to safeguard their mental well-being. Similarly, there may be a need to enhance CMM prevention measures when treating patients with depression to prevent a cycle in which mental and physical health issues exacerbate each other. In summary, prioritizing integrated, person-centered care that addresses both CMM and depression is essential. This approach can help alleviate the burden of these conditions, enhance physical and mental health, and mitigate adverse health outcomes during middle and old age.

### Study Limitations

First, the information of CMM, including diabetes, heart diseases, and stroke, was self-reported, and this was subject to recall bias. However, previous studies have shown that self-reported CMDs have good credibility.^48^, ^49^, ^50^, ^51^ Second, the included participants were from high-income countries or upper-middle income countries. Whether the bidirectional associations between CMM and depression apply to populations from lower-middle and low-income countries warrants further investigation. Third, the information of CMM symptoms and severity, and the diagnosis, medication, and subtypes of depression were unavailable, which could influence observed associations. Last, we included 4 waves of each cohort with only 8-year follow-up to capture the information of exposures and outcomes, which is subject to reverse causality effects. Therefore, future population-based studies with long-term follow-up are needed. Finally, because of data availability limitations and population differences among the included cohorts, measurement of depressive symptoms relied on study-specific multi-item instruments, rather than the same measures across all cohorts. Although we used predefined cutoffs for defining caseness, and all instruments were validated,^52^, ^53^, ^54^, ^55^ the variations in measurement tools across cohorts may have contributed to increased heterogeneity in cohort-specific results. Ideally, future research should use similar protocols and consistent cutoff thresholds across all included cohorts to confirm the validity of the findings.^56^^,^^57^

## Conclusions

CMM and depression showed bidirectional associations, with the onset of either condition significantly increasing the risk of developing the other. Our findings underscore the importance of holistic and integrated approaches that address both CMM and depression to enhance health outcomes and mitigate the reciprocal impact of these conditions among middle-aged and older populations.

Perspectives **COMPETENCY IN MEDICAL KNOWLEDGE:** CMM and depression were bidirectionally associated, and the mediated effects of lifestyle factors were larger in the depression-lifestyle-CMM pathway than in the CMM-lifestyle-depression pathway.

**TRANSLATIONAL OUTLOOK:** Our findings underscore the importance of holistic and integrated approaches that address both CMM and depression to enhance health outcomes and mitigate the reciprocal impact of these conditions.

## Funding Support and Author Disclosures

Dr Xu was supported by the Hundred Talents Program Research Initiation Fund from Zhejiang University and the Fundamental Research Funds for the Central Universities. Mr Kivimäki was supported by the Wellcome Trust (grant 221854/Z/20/Z), the UK Medical Research Council (grant MR/S011676/1 and MR/R024227/1), the National Institute on Aging (grant R01AG056477), and the Academy of Finland (grant 350426). The funder of the study had no role in study design, data collection, data analysis, data interpretation, or the writing of the manuscript. All other authors have reported that they have no relationships relevant to the contents of this paper to disclose.
