# Supplementary material for: Bidirectional Associations Between Cardiometabolic Multimorbidity and Depression and Mediation of Lifestyles: A Multicohort Study
Source: JACC Asia. 2024 Aug 13;4(9):657–71. doi: 10.1016/j.jacasi.2024.06.004 (PMC11450941; doi:10.1016/j.jacasi.2024.06.004)
Supplement: Supplemental Material [file mmc1.docx]

**Supplemental materials**

**Supplemental method 1**

**Table 1** Countries, waves, time periods and sample sizes included in present analyses according to studies.

**Table 2** Harmonized strategies for key variables included in present analyses.

**Table 3** Generalized estimating equation analyses of the bidirectional associations between cardiometabolic multimorbidity and depression.

**Table 4** Subgroup analyses for the association between CMM at baseline and incidence of depression at follow-up by sex and age.

**Table 5** Subgroup analyses for the association between CMM at baseline and incidence of depression at follow-up by educational level.

**Table 6** Subgroup analyses for the association between CMM at baseline and incidence of depression at follow-up by total household wealth.

**Table 7** Statistical characteristics for separate latent class growth models for depression trajectory in CMM-depression analyses (n=26,553).

**Table 8** Odds ratios (ORs) and 95% CIs for the association between CMM at baseline and depression trajectory during follow-up in the CMM-depression analyses.

**Table 9** Subgroup analyses for the association between depression at baseline and the incidence of CMM during follow-up by sex and age.

**Table 10** Subgroup analyses for the association between depression at baseline and CMM during follow-up by educational level.

**Table 11** Subgroup analyses for the association between depression at baseline and CMM during follow-up by total household wealth.

**Figure 1** Meta-analyses of the bidirectional associations between cardiometabolic multimorbidity and depression stratified by sex. (A) CMM-depression analyses in males; (B) CMM-depression analyses in females; (C) Depression-CMM analyses in males; (D) Depression-CMM analyses in females.

**Figure 2** Meta-analyses of the bidirectional associations between cardiometabolic multimorbidity and depression base on Generalized estimating equation analyses. (A) CMM-depression analyses; (B) Depression-CMM analyses.

**Figure 3** Trajectory of depressive symptoms during follow-up. Class 1: persistent symptom-free trajectory; class 2: persistently low trajectory; class 3: increasing trajectory.

**Supplemental method 1**

The depression symptoms were standardized to z scores among individuals who provided information on depression in at least three waves. The z score of depression symptoms was obtained by subtracting the original score by the mean and dividing it by the standard deviation (SD). The depression trajectories were identified using a group-based trajectory model implemented using the PROC TRAJ procedure in SAS. In this procedure, the probabilities for multiple trajectories were estimated to identify the differences between groups of individuals. All available z scores of depression symptoms were used to model the longitudinal depression trajectories. The optimal number and fitted shape of trajectories was identified based on Bayesian information criterion (BIC), Akaike’s information criterion (AIC) and likelihood. Participants were classified into a specific trajectory according to the maximum posteriori probability of assignment. An average posterior probability values of 0.70 or higher was considered a good fit for their most likely class.

**Table 1** Countries, waves, time periods and sample sizes included in present analyses according to studies.

| Study | Country | Wave | Time period | Sample size | |
| --- | --- | --- | --- | --- | --- |
|  |  |  |  | The CMM-depression analyses | The depression-CMM analyses |
| CHARLS | China | 1-4 | 2011-2019 | 7228 | 9708 |
| KLoSA | South Korea | 4-7 | 2012-2019 | 4827 | 4976 |
| HRS | US | 11-14 | 2012-2019 | 12635 | 9980 |
| ELSA | UK | 6-9 | 2012-2019 | 5828 | 4884 |
| SHARE | Austria40 | 5-8 | 2013-2020 | 2534 | 2357 |
|  | Belgium56 | 5-8 | 2013-2020 | 3092 | 3244 |
|  | Czech Republic203 | 5-8 | 2013-2020 | 3340 | 2926 |
|  | Denmark208 | 5-8 | 2013-2020 | 2800 | 2649 |
|  | Estonia233 | 5-8 | 2013-2020 | 2985 | 2889 |
|  | France250 | 5-8 | 2013-2020 | 2163 | 2406 |
|  | Germany276 | 5-8 | 2013-2020 | 3281 | 3218 |
|  | Israel376 | 5-8 | 2013-2020 | 1363 | 1188 |
|  | Italy380 | 5-8 | 2013-2020 | 2424 | 2822 |
|  | Luxembourg442 | 5-8 | 2013-2020 | 835 | 928 |
|  | Netherlands528 | 5-8 | 2013-2020 | 1430 | 1320 |
|  | Slovenia705 | 5-8 | 2013-2020 | 1847 | 1677 |
|  | Spain724 | 5-8 | 2013-2020 | 3553 | 3767 |
|  | Sweden752 | 5-8 | 2013-2020 | 2879 | 2697 |
|  | Switzerland756 | 5-8 | 2013-2020 | 2144 | 2102 |

CMM, cardiometabolic multimorbidity; CHARLS, China Health and Retirement Longitudinal Study; KLoSA, Korean Longitudinal Study of Aging; HRS, US Health and Retirement Study; ELSA, English Longitudinal Study on Ageing; SHARE, Survey of Health, Ageing and Retirement in Europe.

**Table 2** Harmonized strategies for key variables included in present analyses.

| **Variables** | **Harmonized values** | **Measurements in five studies** | | | | |
| --- | --- | --- | --- | --- | --- | --- |
|  |  | **CHARLS** | **KLoSA** | **HRS** | **ELSA** | **SHARE** |
| **Diabetes** | Yes | Self-report of diabetes or high blood sugar | | | | |
|  | No | Otherwise | | | | |
| **Heart diseases** | Yes | Self-report of heart attack, coronary heart disease, angina, congestive heart failure, or other heart problems | Self-report of heart attack, coronary heart disease, angina, congestive heart failure | Self-report of heart attack, coronary heart disease, angina, congestive heart failure, or other heart problems | Self-report of angina, a heart attack (including myocardial infarction or coronary thrombosis), congestive heart failure, a heart murmur, an abnormal heart rhythm, or any other heart trouble | Self-report of heart attack, including myocardial infarction or coronary thrombosis, or any other heart problem, including congestive heart failure |
|  | No | Otherwise | Otherwise | Otherwise | Otherwise | Otherwise |
| **Stroke** | Yes | Self-report of stroke | Self-report of stroke or possible ischemic attack | Self-report of stroke or transient ischemic attack (TIA) | Self-report of stroke or cerebrovascular disease | Self-report of stroke or cerebrovascular disease |
|  | No | Otherwise | Otherwise | Otherwise | Otherwise | Otherwise |
| **Depression** | Yes | CESD-10 score ≥10 | | CESD-8 score ≥3 | CESD-8 score ≥4 | EURO-D score ≥4 |
|  | No | CESD-10 score <10 | | CESD-8 score <3 | CESD-8 score <4 | EURO-D score <4 |
| **Educational level** | Primary | Less than upper secondary education | | | | |
|  | Secondary | Upper secondary & vocational training | | | | |
|  | Tertiary | Tertiary education | | | | |
| **Total household wealth** | Q1 (lowest) | The sum of all wealth components (including residence, vehicles, saving accounts, etc.) minus other debts at the couple level (the respondent and spouse, if any) in local currencies | | | | |
|  | Q2 |  |  |  |  |  |
|  | Q3 |  |  |  |  |  |
|  | Q4 (highest) |  |  |  |  |  |
| **Smoking status** | Yes | Smokes at the present time | | | | |
|  | No | Does not smoke at the present time | | | | |
| **Drinking status** | Weekly drinking or more | The frequency of drinking behaviour during the last year: Once a week/2 to 3 days a week/4 to 6 days a week/Daily/ Twice a day / More than twice a day | The frequency of drinking last year: none or less than once a month / one to several times a month | The frequency of drinking:1-7 days/week | | Whether t drinks weekly or has had an alcoholic drink during the last 7 days: Yes |
|  | Less than weekly drinking | None or doesn’t drink / Once a month / 2 to 3 days a month | One to several times a week / most day of the week / Every day of the week | 0 day/week | | No |
| **Physical activity** | Yes | The number of days of vigorous/moderate physical activity for at least 10 minutes every week: 1-7 days | Works out or exercises more than once a week: Yes | Frequency of taking part in vigorous/moderate physical activity: everday / more than once a week / once a week / one to three times a month | | |
|  | No | None | No | Hardly ever or never taking part in vigorous/moderate physical activity | | |
| **Hypertension** | Yes | The respondent reported having high blood pressure | | | | |
|  | No | The respondent reported having no high blood pressure | | | | |

**Table 3** Generalized estimating equation analyses of the bidirectional associations between cardiometabolic multimorbidity and depression.

|  | **RR (95% CI)** | | | | | | | |  |
| --- | --- | --- | --- | --- | --- | --- | --- | --- | --- |
|  | **Model 1^*^** | | **Model 2^#^** | | **Model 3^†^** | | **Model 4^‡^** | |  |
| ***The CMM-depression analyses*** |  | |  | |  | |  | |  |
| **CMM status → Depression** | | | | | | | | |  |
| No | 1.00 (REF) | | 1.00 (REF) | | 1.00 (REF) | | 1.00 (REF) | |  |
| Yes | 1.87 (1.80-1.95) | | 1.76 (1.69-1.84) | | 1.68 (1.59-1.78) | | 1.54 (1.46-1.62) | |  |
| **CMD status → Depression** | | | | | | | | |  |
| Free of CMD | 1.00 (REF) | | 1.00 (REF) | | 1.00 (REF) | | 1.00 (REF) | |  |
| Single CMD | 1.50 (1.45-1.55) | | 1.45 (1.41-1.50) | | 1.43 (1.37-1.50) | | 1.34 (1.29-1.40) | |  |
| CMM | 2.19 (2.10-2.28) | | 2.05 (1.96-2.14) | | 1.98 (1.87-2.10) | | 1.78 (1.68-1.89) | |  |
| ***The depression-CMM analyses*** | |  | |  | |  | |  | |
| **Depression → CMM status** | | | | | | | | |  |
| No | 1.00 (REF) | | 1.00 (REF) | | 1.00 (REF) | | 1.00 (REF) | |  |
| Yes | 1.86 (1.67-2.08) | | 1.82 (1.62-2.04) | | 1.66 (1.44-1.92) | | 1.49 (1.30-1.71) | |  |
| **Depression → CMD status** | | | | | | | | |  |
| Free of CMD | 1.00 (REF) | | 1.00 (REF) | | 1.00 (REF) | | 1.00 (REF) | |  |
| Single CMD | 1.36 (1.31-1.41) | | 1.34 (1.29-1.39) | | 1.27 (1.21-1.34) | | 1.20 (1.14-1.26) | |  |
| CMM | 1.96 (1.75-2.18) | | 1.91 (1.70-2.14) | | 1.72 (1.49-1.99) | | 1.53 (1.33-1.76) | |  |

^*^adjusted for age, sex and study.

^#^adjusted for age, sex, study, marital status, educational level and total household wealth.

**^†^**adjusted for age, sex, study, marital status, educational level, total household wealth, body mass index (BMI), physical activity, alcohol consumption and smoking status.

**^‡^**adjusted for age, sex, study, marital status, educational level, total household wealth, body mass index (BMI), physical activity, alcohol consumption, smoking status, history of hypertension, cancer and lung diseases, and medication of hypertension and lung diseases.

Abbreviations: CI, confidence interval; CMM, cardiometabolic multimorbidity; CMD, cardiometabolic disease; RR, relative risk.

**Table 4** Subgroup analyses for the association between CMM at baseline and incidence of depression at follow-up by sex and age.

|  | **Cases/n** | **HR (95% CI)** | **Cases/n** | **HR (95% CI)** |
| --- | --- | --- | --- | --- |
|  | **Men** | | **Women** | |
| **CMM status → depression** |  |  |  |  |
| No | 5953/30140 | 1.00 (REF) | 9550/33834 | 1.00 (REF) |
| Yes | 520/1805 | 1.42 (1.29-1.56) | 573/1409 | 1.39 (1.27-1.52) |
| **CMD status → depression** |  |  |  |  |
| None | 4260/22634 | 1.00 (REF) | 7270/27258 | 1.00 (REF) |
| Single CMD | 1693/7506 | 1.24 (1.16-1.32) | 2280/6576 | 1.24 (1.17-1.30) |
| CMM | 520/1805 | 1.55 (1.40-1.71) | 573/1409 | 1.50 (1.37-1.65) |
| **Number of CMDs → depression** |  |  |  |  |
| 0 | 4260/22634 | 1.00 (REF) | 7270/27258 | 1.00 (REF) |
| 1 | 1693/7506 | 1.24 (1.16-1.32) | 2280/6576 | 1.24 (1.17-1.31) |
| 2 | 464/1618 | 1.54 (1.38-1.71) | 516/1284 | 1.49 (1.35-1.65) |
| 3 | 56/187 | 1.66 (1.26-2.18) | 57/125 | 1.56 (1.18-2.05) |
| **CMD combinations → depression** |  |  |  |  |
| None | 4260/22634 | 1.00 (REF) | 7270/27258 | 1.00 (REF) |
| Diabetes only | 698/3259 | 1.16 (1.06-1.26) | 1027/3050 | 1.23 (1.14-1.32) |
| Heart diseases only | 817/3514 | 1.31 (1.20-1.42) | 1007/2847 | 1.23 (1.15-1.33) |
| Stroke only | 178/733 | 1.33 (1.14-1.56) | 246/679 | 1.31 (1.14-1.50) |
| Diabetes and heart diseases | 291/1038 | 1.48 (1.31-1.69) | 320/803 | 1.52 (1.35-1.72) |
| Diabetes and stroke | 63/223 | 1.59 (1.22-2.06) | 74/168 | 1.55 (1.21-1.99) |
| Heart diseases and stroke | 110/357 | 1.66 (1.36-2.03) | 122/313 | 1.39 (1.15-1.68) |
| Diabetes, heart diseases and stroke | 56/187 | 1.66 (1.27-2.18) | 57/125 | 1.56 (1.18-2.05) |
|  | **< 65 years** | | **≥ 65 years** | |
| **CMM status → depression** |  |  |  |  |
| No | 8093/34741 | 1.00 (REF) | 7410/29233 | 1.00 (REF) |
| Yes | 292/869 | 1.40 (1.23-1.60) | 801/2345 | 1.45 (1.34-1.57) |
| **CMD status → depression** |  |  |  |  |
| None | 6634/29512 | 1.00 (REF) | 4896/20380 | 1.00 (REF) |
| Single CMD | 1459/5229 | 1.28 (1.20-1.37) | 2514/8853 | 1.24 (1.18-1.31) |
| CMM | 292/869 | 1.51 (1.32-1.72) | 801/2345 | 1.58 (1.46-1.71) |
| **Number of CMDs → depression** |  |  |  |  |
| 0 | 6634/29512 | 1.00 (REF) | 4896/20380 | 1.00 (REF) |
| 1 | 1459/5229 | 1.28 (1.20-1.37) | 2514/8853 | 1.24 (1.18-1.31) |
| 2 | 264/806 | 1.46 (1.28-1.68) | 716/2096 | 1.58 (1.46-1.72) |
| 3 | 28/63 | 2.00 (1.37-2.94) | 85/249 | 1.56 (1.24-1.95) |
| **CMD combinations → depression** |  |  |  |  |
| None | 6634/29512 | 1.00 (REF) | 4896/20380 | 1.00 (REF) |
| Diabetes only | 709/2722 | 1.22 (1.12-1.32) | 1016/3587 | 1.17 (1.09-1.26) |
| Heart diseases only | 603/2047 | 1.32 (1.20-1.46) | 1221/4314 | 1.29 (1.21-1.38) |
| Stroke only | 147/460 | 1.48 (1.24-1.77) | 277/952 | 1.28 (1.13-1.45) |
| Diabetes and heart diseases | 171/545 | 1.42 (1.20-1.68) | 440/1296 | 1.56 (1.41-1.73) |
| Diabetes and stroke | 46/132 | 1.42 (1.02-1.98) | 91/259 | 1.67 (1.34-2.07) |
| Heart diseases and stroke | 47/129 | 1.66 (1.23-2.26) | 185/541 | 1.59 (1.37-1.86) |
| Diabetes, heart diseases and stroke | 28/63 | 2.00 (1.37-2.93) | 85/249 | 1.56 (1.24-1.95) |

The subgroup analyses by sex were adjusted for age, study, marital status, educational level, total household wealth, body mass index (BMI), physical activity, alcohol consumption, smoking status, and history of hypertension, cancer and lung diseases. The subgroup analyses by age were adjusted for sex, study, marital status, educational level, total household wealth, body mass index (BMI), physical activity, alcohol consumption, smoking status, history of hypertension, cancer and lung diseases and medication of hypertension and lung diseases.

Abbreviations: CMD, cardiometabolic disease; CMM, cardiometabolic multimorbidity; HR, hazard ratio; CI, confidence interval.

**Table 5** Subgroup analyses for the association between CMM at baseline and incidence of depression at follow-up by educational level.

|  | **Primary** | | **Secondary** | | **Tertiary** | |
| --- | --- | --- | --- | --- | --- | --- |
|  | **Cases/n** | **HR (95% CI)** | **Cases/n** | **HR (95% CI)** | **Cases/n** | **HR (95% CI)** |
| **CMM status → depression** |  |  |  |  |  |  |
| No | 6895/21687 | 1.00 (REF) | 5509/24935 | 1.00 (REF) | 3099/17352 | 1.00 (REF) |
| Yes | 488/1239 | 1.27 (1.14-1.40) | 383/1192 | 1.52 (1.36-1.70) | 222/783 | 1.52 (1.32-1.76) |
| **CMD status → depression** |  |  |  |  |  |  |
| None | 5142/16732 | 1.00 (REF) | 4071/19526 | 1.00 (REF) | 2317/13634 | 1.00 (REF) |
| Single CMD | 1753/4955 | 1.20 (1.12-1.27) | 1438/5409 | 1.31 (1.23-1.40) | 782/3718 | 1.18 (1.08-1.29) |
| CMM | 488/1239 | 1.35 (1.22-1.50) | 383/1192 | 1.69 (1.51-1.89) | 222/783 | 1.62 (1.40-1.88) |
| **Number of CMDs → depression** |  |  |  |  |  |  |
| 0 | 5142/16732 | 1.00 (REF) | 4071/19526 | 1.00 (REF) | 2317/13634 | 1.00 (REF) |
| 1 | 1753/4955 | 1.20 (1.12-1.27) | 1438/5409 | 1.31 (1.23-1.40) | 782/3718 | 1.18 (1.08-1.29) |
| 2 | 441/1117 | 1.36 (1.22-1.52) | 337/1070 | 1.65 (1.46-1.86) | 202/715 | 1.62 (1.39-1.89) |
| 3 | 47/122 | 1.29 (0.95-1.75) | 46/122 | 2.06 (1.52-2.78) | 20/68 | 1.69 (1.07-2.66) |
| **CMD combinations → depression** |  |  |  |  |  |  |
| None | 5142/16732 | 1.00 (REF) | 4071/19526 | 1.00 (REF) | 2317/13634 | 1.00 (REF) |
| Diabetes only | 844/2339 | 1.24 (1.15-1.35) | 566/2333 | 1.22 (1.11-1.34) | 315/1637 | 1.03 (0.91-1.17) |
| Heart diseases only | 739/2112 | 1.14 (1.05-1.25) | 703/2522 | 1.37 (1.25-1.50) | 382/1727 | 1.32 (1.17-1.47) |
| Stroke only | 170/504 | 1.19 (1.01-1.40) | 169/554 | 1.48 (1.26-1.74) | 85/354 | 1.30 (1.04-1.62) |
| Diabetes and heart diseases | 279/711 | 1.36 (1.19-1.55) | 213/676 | 1.69 (1.45-1.95) | 119/454 | 1.53 (1.26-1.85) |
| Diabetes and stroke | 73/164 | 1.65 (1.28-2.13) | 44/149 | 1.54 (1.12-2.12) | 20/78 | 1.35 (0.86-2.10) |
| Heart diseases and stroke | 89/242 | 1.20 (0.96-1.50) | 80/245 | 1.62 (1.28-2.04) | 63/183 | 1.97 (1.52-2.55) |
| Diabetes, heart diseases and stroke | 47/122 | 1.29 (0.95-1.75) | 46/122 | 2.06 (1.52-2.78) | 20/68 | 1.68 (1.07-2.66) |

The models were adjusted for age, sex, study, marital status, total household wealth, body mass index (BMI), physical activity, alcohol consumption, smoking status, history of hypertension, cancer and lung diseases and medication of hypertension and lung diseases.

Abbreviations: CMD, cardiometabolic disease; CMM, cardiometabolic multimorbidity; HR, hazard ratio; CI, confidence interval.

**Table 6** Subgroup analyses for the association between CMM at baseline and incidence of depression at follow-up by total household wealth.

|  | **Lower than median** | | **Higher than median** | |
| --- | --- | --- | --- | --- |
|  | **Cases/n** | **HR (95% CI)** | **Cases/n** | **HR (95% CI)** |
| **CMM status → depression** |  |  |  |  |
| No | 8338/30662 | 1.00 (REF) | 6465/31385 | 1.00 (REF) |
| Yes | 716/1970 | 1.33 (1.23-1.45) | 363/1216 | 1.56 (1.40-1.75) |
| **CMD status → depression** |  |  |  |  |
| None | 5951/22981 | 1.00 (REF) | 4979/25239 | 1.00 (REF) |
| Single CMD | 2387/7681 | 1.23 (1.17-1.30) | 1486/6146 | 1.24 (1.17-1.33) |
| CMM | 716/1970 | 1.45 (1.33-1.58) | 363/1216 | 1.69 (1.50-1.89) |
| **Number of CMDs → depression** |  |  |  |  |
| 0 | 5951/22981 | 1.00 (REF) | 4979/25239 | 1.00 (REF) |
| 1 | 2387/7681 | 1.23 (1.17-1.30) | 1486/6146 | 1.24 (1.17-1.33) |
| 2 | 640/1771 | 1.45 (1.33-1.58) | 326/1104 | 1.66 (1.47-1.87) |
| 3 | 76/199 | 1.49 (1.17-1.88) | 37/112 | 1.96 (1.40-2.76) |
| **CMD combinations → depression** |  |  |  |  |
| None | 5951/22981 | 1.00 (REF) | 4979/25239 | 1.00 (REF) |
| Diabetes only | 1109/3626 | 1.22 (1.14-1.31) | 589/2596 | 1.15 (1.05-1.26) |
| Heart diseases only | 1040/3296 | 1.25 (1.17-1.35) | 721/2915 | 1.28 (1.18-1.40) |
| Stroke only | 238/759 | 1.21 (1.06-1.39) | 176/635 | 1.48 (1.26-1.73) |
| Diabetes and heart diseases | 393/1129 | 1.42 (1.27-1.58) | 209/695 | 1.67 (1.44-1.94) |
| Diabetes and stroke | 95/251 | 1.61 (1.31-1.99) | 37/134 | 1.51 (1.07-2.14) |
| Heart diseases and stroke | 152/391 | 1.43 (1.20-1.70) | 80/275 | 1.71 (1.36-2.14) |
| Diabetes, heart diseases and stroke | 76/199 | 1.49 (1.17-1.88) | 37/112 | 1.96 (1.40-2.75) |

The models were adjusted for age, sex, study, marital status, educational level, body mass index (BMI), physical activity, alcohol consumption, smoking status, history of hypertension, cancer and lung diseases and medication of hypertension and lung diseases.

Abbreviations: CMD, cardiometabolic disease; CMM, cardiometabolic multimorbidity; HR, hazard ratio; CI, confidence interval.

**Table 7** Statistical characteristics for separate latent class growth models for depression trajectory in CMM-depression analyses (n=26,553).

| No. Latent classes | Polynomial degree | BIC | AIC | Likelihood | Group precents | Mean posterior probabilities | Posterior probabilities >0.7 (%) |
| --- | --- | --- | --- | --- | --- | --- | --- |
| 1 | Quadratic | -110917.6 | -110901.3 | -110897.3 | 100 |  |  |
| 2 | Linear | -104854.4 | -104829.9 | -104829.9 | 83.70/16.30 | 0.96/0.87 | 95.83/81.65 |
| 2 | Quadratic | -104815.0 | -104782.2 | -104774.2 | 84.33/15.67 | 0.96/0.87 | 95.61/83.51 |
| 2 | Cubic | -104825.2 | -104784.2 | -104774.2 | 84.33/15.67 | 0.96/0.87 | 95.61/83.51 |
| **3** | **Linear** | -102410.1 | -102373.3 | -102364.3 | **67.42/26.26/6.32** | **0.93/0.81/0.88** | **91.77/71.28/81.70** |
| 3 | Quadratic | -102131.9 | -102082.8 | -102070.8 | 65.68/26.45/7.87 | 0.91/0.82/0.89 | 86.58/71.86/87.27 |
| 3 | Cubic | -102147.2 | -102085.8 | -102070.8 | 65.68/26.45/7.87 | 0.91/0.82/0.89 | 86.588/71.86/87.27 |

CMM, cardiometabolic multimorbidity; BIC, Bayesian information criteria; AIC, Akaike information criterion.

**Table 8** Odds ratios (ORs) and 95% CIs for the association between CMM at baseline and depression trajectory during follow-up in the CMM-depression analyses.

|  | **Persistently free** | | **Persistently low** | | **Increasing** | |
| --- | --- | --- | --- | --- | --- | --- |
|  | **Cases/n** | **OR (95% CI)** | **Cases/n** | **OR (95% CI)** | **Cases/n** | **OR (95% CI)** |
| **CMM status → depression** |  |  |  |  |  |  |
| No | 17260/25396 | Ref | 6585/25396 | 1.00 (REF) | 1551/25396 | 1.00 (REF) |
| Yes | 641/1157 | Ref | 389/1157 | 1.48 (1.28-1.70) | 127/1157 | 1.72 (1.38-2.15) |
| **CMD status → depression** |  |  |  |  |  |  |
| None | 13626/19744 | Ref | 5010/19744 | 1.00 (REF) | 1108/19744 | 1.00 (REF) |
| Single CMD | 3634/5652 | Ref | 1575/5652 | 1.18 (1.09-1.28) | 443/5652 | 1.47 (1.28-1.68) |
| CMM | 641/1157 | Ref | 389/1157 | 1.57 (1.36-1.82) | 127/1157 | 2.01 (1.60-2.53) |
| **Number of CMDs → depression** |  |  |  |  |  |  |
| 0 | 13626/19744 | Ref | 5010/19744 | 1.00 (REF) | 1108/19744 | 1.00 (REF) |
| 1 | 3634/5652 | Ref | 1575/5652 | 1.18 (1.09-1.28) | 443/5652 | 1.47 (1.28-1.68) |
| 2 | 599/1067 | Ref | 353/1067 | 1.52 (1.31-1.77) | 115/1067 | 1.93 (1.52-2.46) |
| 3 | 42/90 | Ref | 36/90 | 2.24 (1.39-3.60) | 12/90 | 3.07 (1.57-6.02) |
| **CMD combinations → depression** |  |  |  |  |  |  |
| None | 13626/19744 | Ref | 5010/19744 | 1.00 (REF) | 1108/19744 | 1.00 (REF) |
| Diabetes only | 1702/2639 | Ref | 744/2639 | 1.14 (1.03-1.27) | 193/2639 | 1.33 (1.11-1.59) |
| Heart diseases only | 1654/2509 | Ref | 656/2509 | 1.12 (1.01-1.26) | 199/2509 | 1.47 (1.22-1.77) |
| Stroke only | 278/504 | Ref | 175/504 | 1.71 (1.39-2.10) | 51/504 | 2.34 (1.69-3.23) |
| Diabetes and heart diseases | 404/715 | Ref | 234/715 | 1.56 (1.30-1.87) | 77/715 | 1.98 (1.49-2.63) |
| Diabetes and stroke | 73/140 | Ref | 52/140 | 1.65 (1.12-2.42) | 15/140 | 2.07 (1.14-3.75) |
| Heart diseases and stroke | 122/212 | Ref | 67/212 | 1.33 (0.96-1.84) | 23/212 | 1.68 (1.01-2.79) |
| Diabetes, heart diseases and stroke | 42/90 | Ref | 36/90 | 2.24 (1.39-3.60) | 12/90 | 3.06 (1.56-6.00) |

The models were adjusted for age, sex, study, marital status, educational level, total household wealth, body mass index (BMI), physical activity, alcohol consumption, smoking status, history of hypertension, cancer and lung diseases, and medication of hypertension and lung diseases.

Abbreviations: CMD, cardiometabolic disease; CMM, cardiometabolic multimorbidity; OR, odd ratio; CI, confidence interval.

**Table 9** Subgroup analyses for the association between depression at baseline and the incidence of CMM during follow-up by sex and age.

|  | **Cases/n** | **HR (95% CI)** | **Cases/n** | **HR (95% CI)** |
| --- | --- | --- | --- | --- |
|  | **Men** | | **Women** | |
| **Depression → CMM status** |  |  |  |  |
| No | 4399/4557 | 1.00 (REF) | 9820/10116 | 1.00 (REF) |
| Yes | 158/4557 | 1.23 (0.99-1.52) | 296/10116 | 1.28 (1.07-1.54) |
| **Depression → CMD status** |  |  |  |  |
| None | 3472/4557 | 1.00 (REF) | 7975/10116 | 1.00 (REF) |
| Single CMD | 927/4557 | 1.25 (1.15-1.36) | 1845/10116 | 1.32 (1.24-1.41) |
| CMM | 158/4557 | 1.27 (1.03-1.58) | 296/10116 | 1.35 (1.12-1.62) |
| **Depression → Number of CMDs** |  |  |  |  |
| 0 | 3472/4557 | 1.00 (REF) | 7975/10116 | 1.00 (REF) |
| 1 | 927/4557 | 1.25 (1.15-1.36) | 1845/10116 | 1.32 (1.24-1.41) |
| 2 | 144/4557 | 1.27 (1.01-1.58) | 270/10116 | 1.36 (1.13-1.64) |
| 3 | 14/4557 | 1.31 (0.60-2.89) | 26/10116 | 1.10 (0.49-2.49) |
| **Depression → CMD combinations** |  |  |  |  |
| None | 3472/4557 | 1.00 (REF) | 7975/10116 | 1.00 (REF) |
| Diabetes only | 307/4557 | 1.21 (1.05-1.40) | 649/10116 | 1.23 (1.11-1.37) |
| Heart diseases only | 456/4557 | 1.28 (1.13-1.45) | 935/10116 | 1.45 (1.32-1.59) |
| Stroke only | 164/4557 | 1.36 (1.11-1.67) | 261/10116 | 1.29 (1.09-1.54) |
| Diabetes and heart diseases | 60/4557 | 1.09 (0.77-1.54) | 149/10116 | 1.48 (1.13-1.92) |
| Diabetes and stroke | 26/4557 | 1.32 (0.76-2.30) | 38/10116 | 1.56 (0.91-2.66) |
| Heart diseases and stroke | 58/4557 | 1.48 (1.05-2.08) | 83/10116 | 1.16 (0.85-1.59) |
| Diabetes, heart diseases and stroke | 14/4557 | 1.31 (0.60-2.89) | 26/10116 | 1.10 (0.49-2.49) |
|  | **< 65 years** | | **≥ 65 years** | |
| **Depression → CMM status** |  |  |  |  |
| No | 8608/8830 | 1.00 (REF) | 5613/5843 | 1.00 (REF) |
| Yes | 224/8830 | 1.29 (1.04-1.59) | 230/5843 | 1.22 (1.02-1.46) |
| **Depression → CMD status** |  |  |  |  |
| None | 7124/8830 | 1.00 (REF) | 4323/5843 | 1.00 (REF) |
| Single CMD | 1482/8830 | 1.31 (1.22-1.41) | 1290/5843 | 1.25 (1.17-1.35) |
| CMM | 224/8830 | 1.36 (1.10-1.68) | 230/5843 | 1.28 (1.06-1.53) |
| **Depression → Number of CMDs** |  |  |  |  |
| 0 | 7124/8830 | 1.00 (REF) | 4323/5843 | 1.00 (REF) |
| 1 | 1482/8830 | 1.31 (1.22-1.41) | 1290/5843 | 1.25 (1.17-1.35) |
| 2 | 204/8830 | 1.37 (1.10-1.70) | 210/5843 | 1.28 (1.06-1.54) |
| 3 | 20/8830 | 1.19 (0.48-2.97) | 20/5843 | 1.27 (0.62-2.60) |
| **Depression → CMD combinations** |  |  |  |  |
| None | 7124/8830 | 1.00 (REF) | 4323/5843 | 1.00 (REF) |
| Diabetes only | 580/8830 | 1.22 (1.09-1.37) | 376/5843 | 1.17 (1.02-1.33) |
| Heart diseases only | 703/8830 | 1.44 (1.29-1.61) | 688/5843 | 1.33 (1.21-1.47) |
| Stroke only | 199/8830 | 1.38 (1.11-1.71) | 226/5843 | 1.29 (1.09-1.53) |
| Diabetes and heart diseases | 115/8830 | 1.45 (1.08-1.94) | 94/5843 | 1.18 (0.88-1.57) |
| Diabetes and stroke | 36/8830 | 1.41 (0.81-2.47) | 28/5843 | 1.48 (0.87-2.50) |
| Heart diseases and stroke | 53/8830 | 1.16 (0.77-1.74) | 88/5843 | 1.35 (1.01-1.79) |
| Diabetes, heart diseases and stroke | 20/8830 | 1.19 (0.48-2.97) | 20/5843 | 1.27 (0.62-2.60) |

The subgroup analyses by sex were adjusted for age, study, marital status, educational level, total household wealth, body mass index (BMI), physical activity, alcohol consumption, smoking status, history of hypertension, cancer and lung diseases, and medication of hypertension and lung diseases. The subgroup analyses by age were adjusted for sex, study, marital status, educational level, total household wealth, body mass index (BMI), physical activity, alcohol consumption, smoking status, history of hypertension, cancer and lung diseases, and medication of hypertension and lung diseases.

Abbreviations: CMD, cardiometabolic disease; CMM, cardiometabolic multimorbidity; HR, hazard ratio;; CI, confidence interval.

**Table 10** Subgroup analyses for the association between depression at baseline and CMM during follow-up by educational level.

|  | **Primary** | | **Secondary** | | **Tertiary** | |
| --- | --- | --- | --- | --- | --- | --- |
|  | **Cases/n** | **HR (95% CI)** | **Cases/n** | **HR (95% CI)** | **Cases/n** | **HR (95% CI)** |
| **Depression → CMM status** |  |  |  |  |  |  |
| No | 7042/7332 | 1.00 (REF) | 4732/4846 | 1.00 (REF) | 2454/2495 | 1.00 (REF) |
| Yes | 290/7332 | 1.27 (1.05-1.55) | 123/4846 | 1.31 (1.04-1.66) | 41/2495 | 1.12 (0.79-1.59) |
| **Depression → CMD status** |  |  |  |  |  |  |
| None | 5552/7332 | 1.00 (REF) | 3851/4846 | 1.00 (REF) | 2044/2495 | 1.00 (REF) |
| Single CMD | 1490/7332 | 1.21 (1.12-1.31) | 872/4846 | 1.37 (1.25-1.49) | 410/2495 | 1.35 (1.21-1.51) |
| CMM | 290/7332 | 1.35 (1.11-1.64) | 123/4846 | 1.44 (1.14-1.82) | 41/2495 | 1.24 (0.87-1.75) |
| **Depression → Number of CMDs** |  |  |  |  |  |  |
| 0 | 5552/7332 | 1.00 (REF) | 3851/4846 | 1.00 (REF) | 2044/2495 | 1.00 (REF) |
| 1 | 1490/7332 | 1.21 (1.12-1.31) | 872/4846 | 1.37 (1.25-1.49) | 410/2495 | 1.35 (1.21-1.51) |
| 2 | 258/7332 | 1.35 (1.10-1.65) | 117/4846 | 1.35 (1.06-1.72) | 39/2495 | 1.18 (0.83-1.69) |
| 3 | 32/7332 | 1.08 (0.52-2.22) | 6/4846 | 2.01 (0.68-5.93) | 2/2495 | 1.21 (0.25-5.93) |
| **Depression → CMD combinations** |  |  |  |  |  |  |
| None | 5552/7332 | 1.00 (REF) | 3851/4846 | 1.00 (REF) | 2044/2495 | 1.00 (REF) |
| Diabetes only | 522/7332 | 1.14 (1.01-1.30) | 306/4846 | 1.36 (1.18-1.57) | 128/2495 | 1.16 (0.95-1.42) |
| Heart diseases only | 732/7332 | 1.30 (1.16-1.46) | 431/4846 | 1.38 (1.22-1.57) | 228/2495 | 1.53 (1.31-1.78) |
| Stroke only | 236/7332 | 1.19 (0.98-1.45) | 135/4846 | 1.48 (1.18-1.83) | 54/2495 | 1.44 (1.06-1.97) |
| Diabetes and heart diseases | 131/7332 | 1.23 (0.93-1.64) | 64/4846 | 1.58 (1.12-2.23) | 14/2495 | 1.04 (0.57-1.87) |
| Diabetes and stroke | 40/7332 | 1.55 (0.89-2.70) | 16/4846 | 1.14 (0.57-2.28) | 8/2495 | 1.76 (0.77-4.02) |
| Heart diseases and stroke | 87/7332 | 1.48 (1.06-2.09) | 37/4846 | 1.14 (0.76-1.69) | 17/2495 | 1.14 (0.66-1.96) |
| Diabetes, heart diseases and stroke | 32/7332 | 1.08 (0.52-2.22) | 6/4846 | 2.01 (0.68-5.93) | 2/2495 | 1.21 (0.25-5.93) |

The models were adjusted for age, sex, study, marital status, total household wealth, body mass index (BMI), physical activity, alcohol consumption, smoking status, history of hypertension, cancer and lung diseases, and medication of hypertension and lung diseases.

Abbreviations: CMD, cardiometabolic disease; CMM, cardiometabolic multimorbidity; HR, hazard ratio; CI, confidence interval.

**Table 11** Subgroup analyses for the association between depression at baseline and CMM during follow-up by total household wealth.

|  | **Lower than median** | | **Higher than median** | |
| --- | --- | --- | --- | --- |
|  | **Cases/n** | **HR (95% CI)** | **Cases/n** | **HR (95% CI)** |
| **Depression → CMM status** |  |  |  |  |
| No | 8072/8350 | 1.00 (REF) | 2796/2865 | 1.00 (REF) |
| Yes | 278/8350 | 1.19 (1.01-1.40) | 69/2865 | 1.58 (1.20-2.09) |
| **Depression → CMD status** |  |  |  |  |
| None | 6375/8350 | 1.00 (REF) | 2301/2865 | 1.00 (REF) |
| Single CMD | 1697/8350 | 1.33 (1.25-1.42) | 495/2865 | 1.22 (1.11-1.36) |
| CMM | 278/8350 | 1.27 (1.08-1.51) | 69/2865 | 1.57 (1.18-2.08) |
| **Depression → Number of CMDs** |  |  |  |  |
| 0 | 6375/8350 | 1.00 (REF) | 2301/2865 | 1.00 (REF) |
| 1 | 1697/8350 | 1.33 (1.25-1.42) | 495/2865 | 1.22 (1.11-1.36) |
| 2 | 255/8350 | 1.29 (1.09-1.53) | 63/2865 | 1.52 (1.13-2.04) |
| 3 | 23/8350 | 1.01 (0.50-2.03) | 6/2865 | 2.47 (0.83-7.35) |
| **Depression → CMD combinations** |  |  |  |  |
| None | 6375/8350 | 1.00 (REF) | 2301/2865 | 1.00 (REF) |
| Diabetes only | 593/8350 | 1.18 (1.06-1.32) | 173/2865 | 1.30 (1.09-1.54) |
| Heart diseases only | 851/8350 | 1.52 (1.38-1.66) | 243/2865 | 1.22 (1.05-1.40) |
| Stroke only | 253/8350 | 1.40 (1.18-1.66) | 79/2865 | 1.18 (0.92-1.52) |
| Diabetes and heart diseases | 133/8350 | 1.25 (0.98-1.59) | 28/2865 | 1.61 (1.03-2.51) |
| Diabetes and stroke | 37/8350 | 1.47 (0.91-2.37) | 11/2865 | 1.75 (0.86-3.56) |
| Heart diseases and stroke | 85/8350 | 1.28 (0.96-1.70) | 24/2865 | 1.45 (0.91-2.31) |
| Diabetes, heart diseases and stroke | 23/8350 | 1.01 (0.50-2.03) | 6/2865 | 2.47 (0.83-7.35) |

The models were adjusted for age, sex, study, marital status, educational level, body mass index (BMI), physical activity, alcohol consumption, smoking status, history of hypertension, cancer and lung diseases, and medication of hypertension and lung diseases.

Abbreviations: CMD, cardiometabolic disease; CMM, cardiometabolic multimorbidity; HR, hazard ratio; CI, confidence interval.


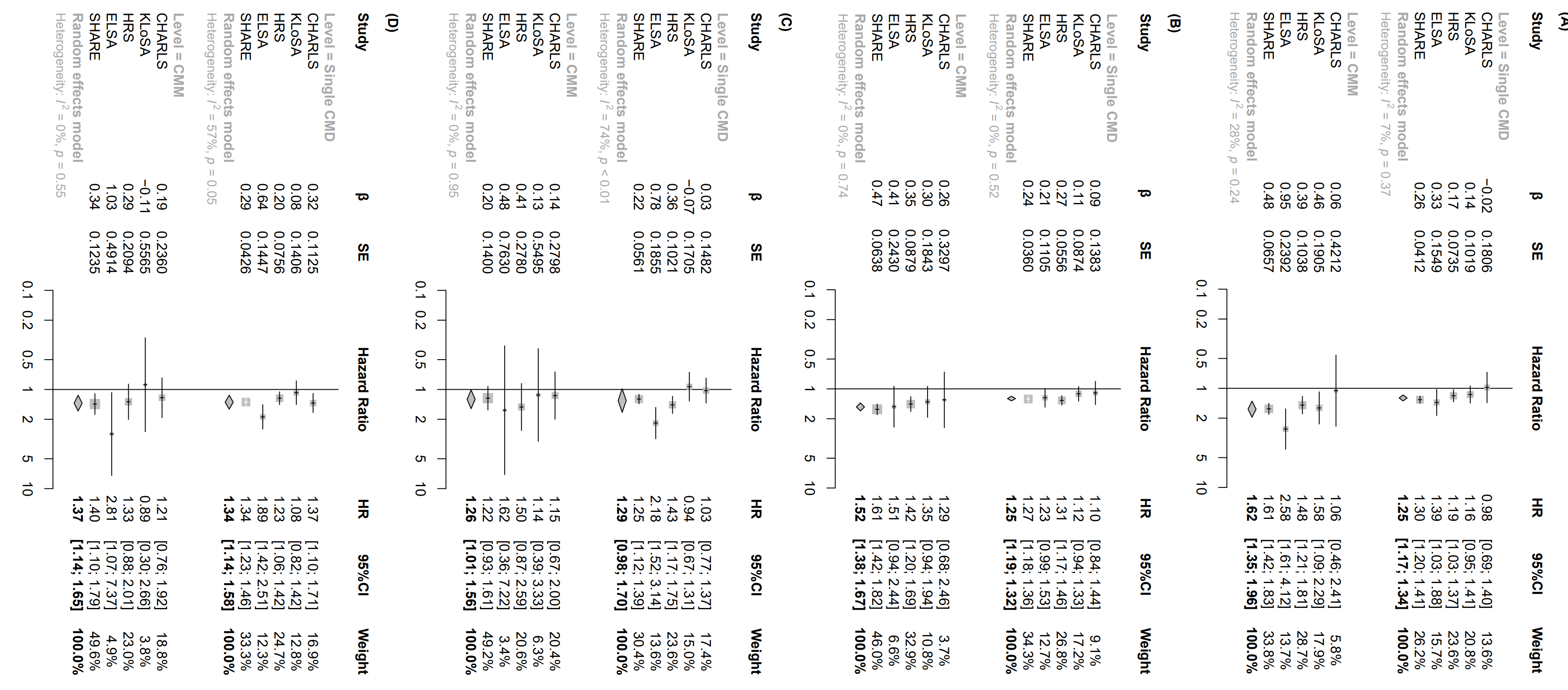


**Figure 1** Meta-analyses of the bidirectional associations between cardiometabolic multimorbidity and depression stratified by sex.

(A) CMM-depression analyses in males; (B) CMM-depression analyses in females; (C) Depression-CMM analyses in males; (D) Depression-CMM analyses in females.


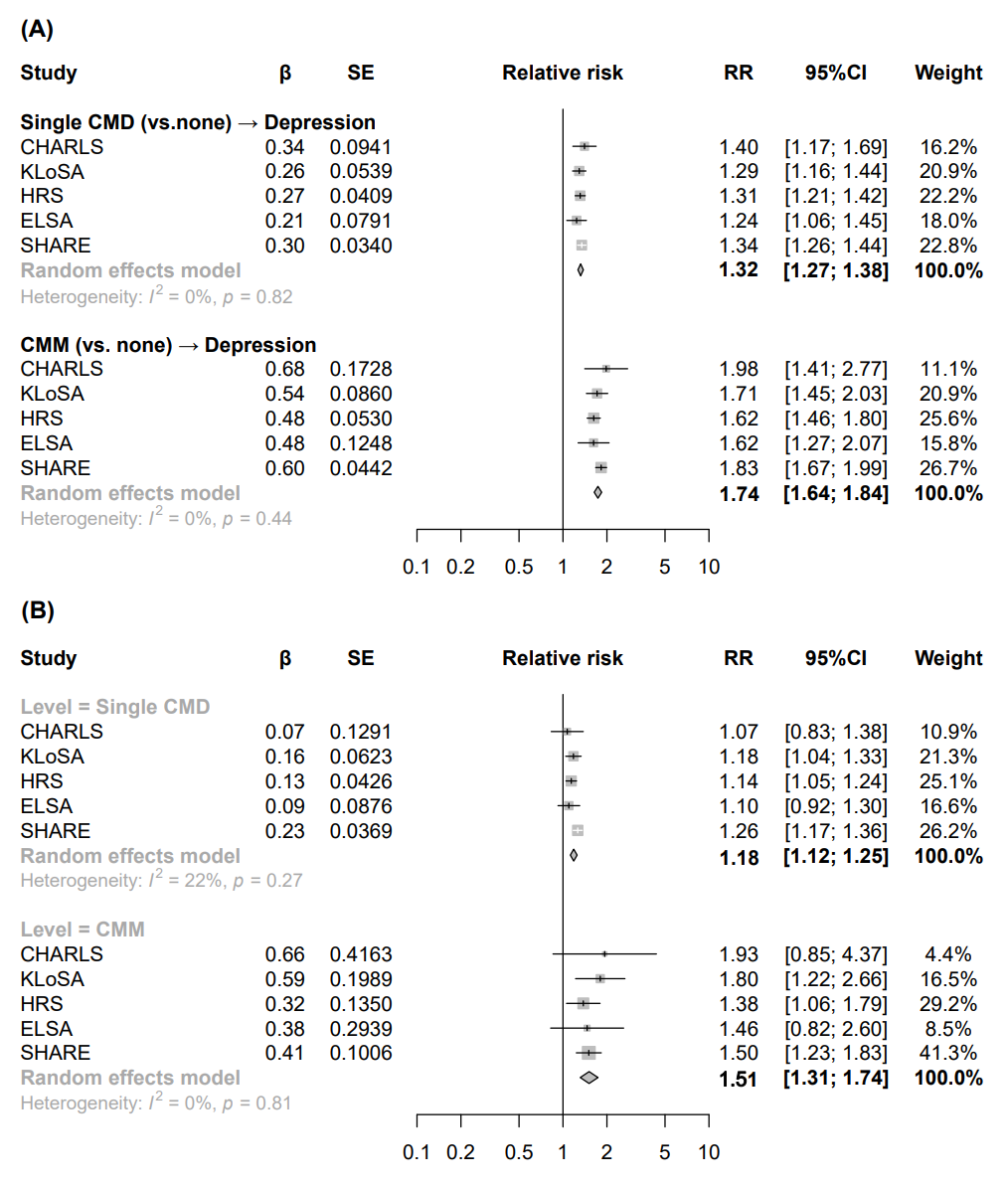


**Figure 2** Meta-analyses of the bidirectional associations between cardiometabolic multimorbidity and depression base on Generalized estimating equation analyses. (A) CMM-depression analyses; (B) Depression-CMM analyses.


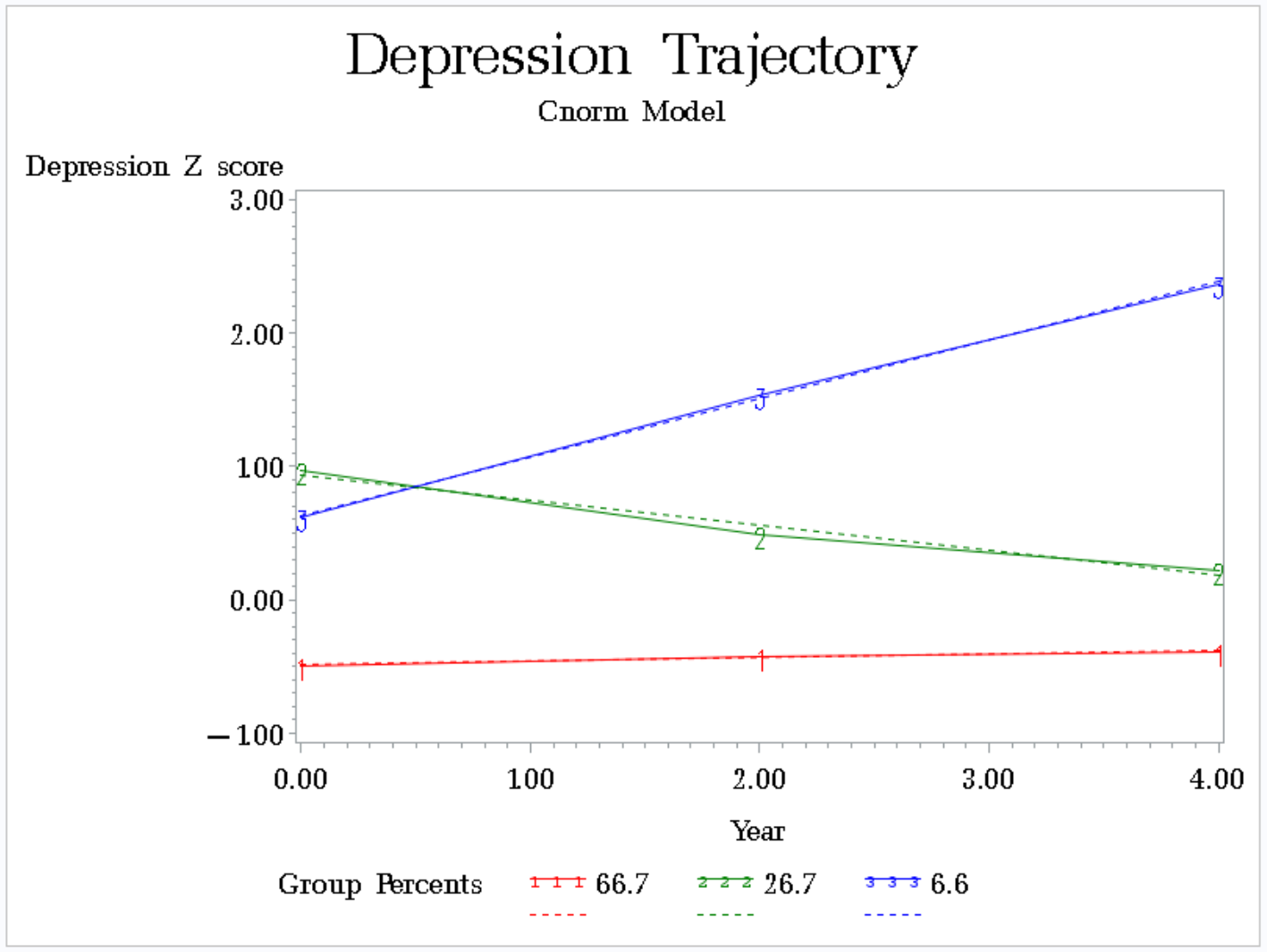


**Figure 3** Trajectory of depressive symptoms during follow-up. Class 1: persistent symptom-free trajectory; class 2: persistently low trajectory; class 3: increasing trajectory.
